# Supplementary material for: Hepatic ROS Mediated Macrophage Activation Is Responsible for Irinotecan Induced Liver Injury
Source: Cells. 2022 Nov 26;11(23):3791. doi: 10.3390/cells11233791 (PMC9739808; doi:10.3390/cells11233791)
Supplement: Supplementary file 1 [file cells-11-03791-s001.zip › cells-1941288-supplementary.pdf]

**Supplementary Materials**

**Figure S1**

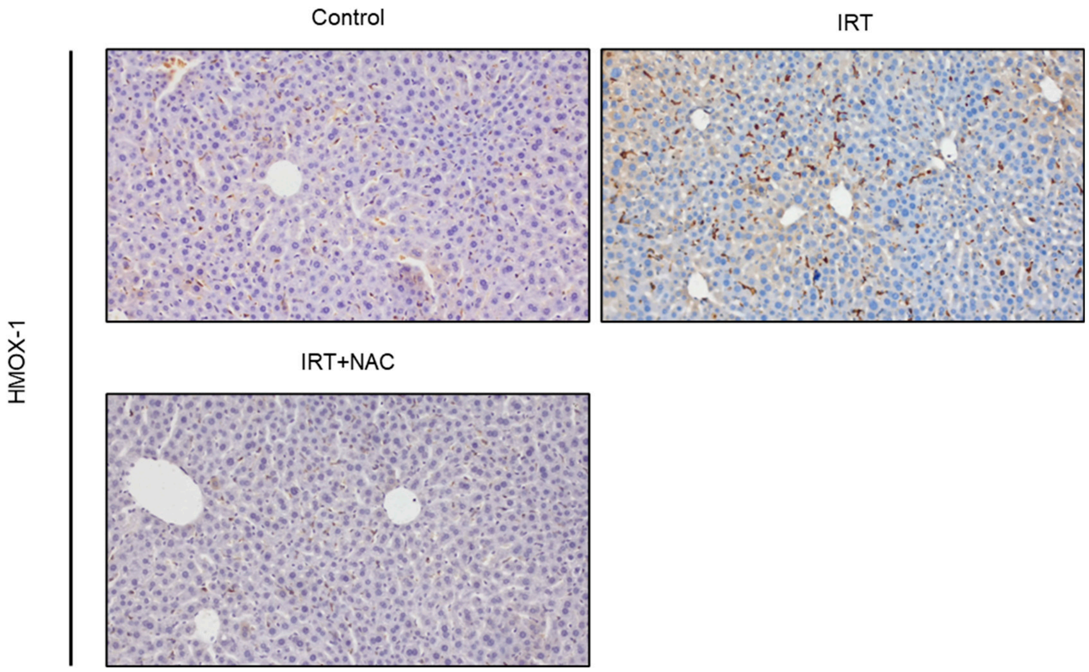


**Figure S1. Irinotecan upregulates HMOX-1 expression in mouse liver.** Mice in the IRT group were injected intraperitoneally with irinotecan hydrochloride, and mice in the NAC group were simultaneously injected intraperitoneally with NAC on the following day, alternately for 2 weeks, and the expression of HMOX-1 in the liver was determined by immunohistochemistry in the control group, IRT group and IRT+NAC group.

**Figure S2**

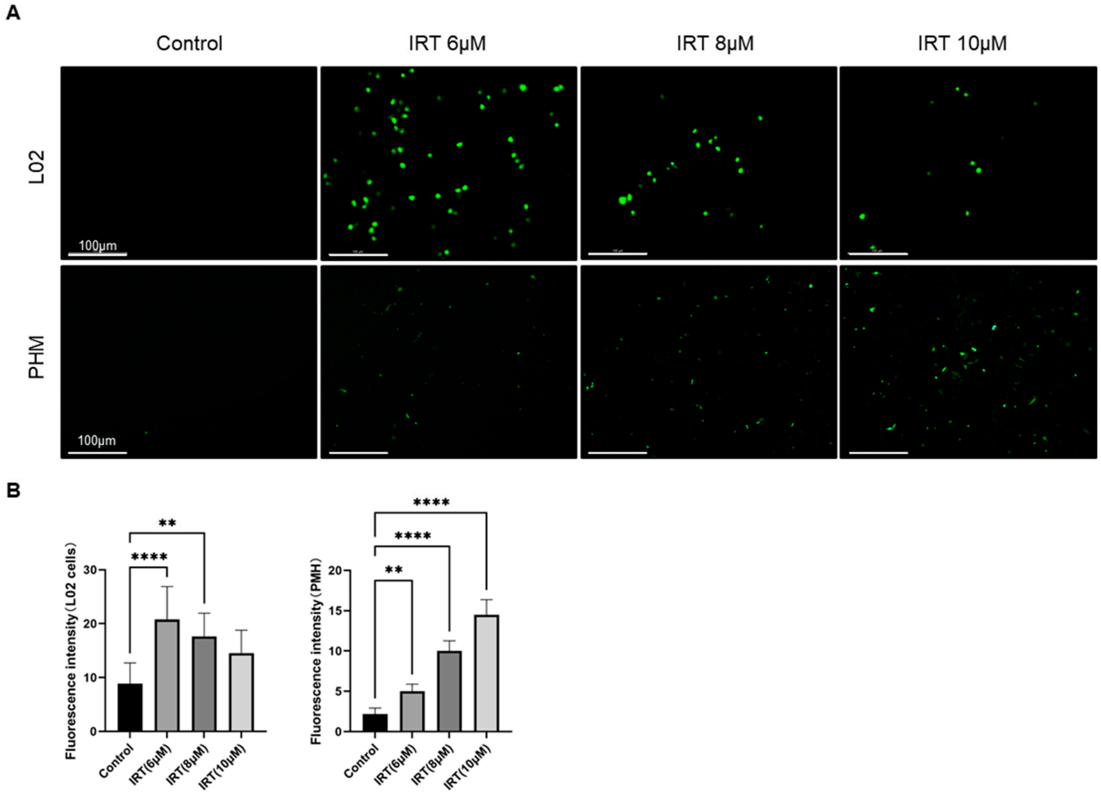


**Figure S2. Measurement of reactive oxygen species after treatment of L02 cells and primary mouse hepatocytes with different concentrations of irinotecan.** L02 cells (3 × $10^5$) and PMH (3 × $10^5$) were cultured in a 24-well plate and incubated with 6 μM,8μM and 10μM of irinotecan for 24 h, and then ROS levels were measured. (**A**) Determination of ROS activity in L02 cells and PMH after different concentrations of irinotecan treatment. (**B**) Quantitative analysis of fluorescence intensity of reactive oxygen species in L02 cells and PMH after treatment with different concentrations of irinotecan. Data are reported as mean ± SD, and each graph is representative of at least 3 independent experiments. Comparison of values was performed by One-way ANOVA for unpaired data. *p <0.05; **p <0.01; ***p <0.001; ****p <0.0001.

**Figure S3**

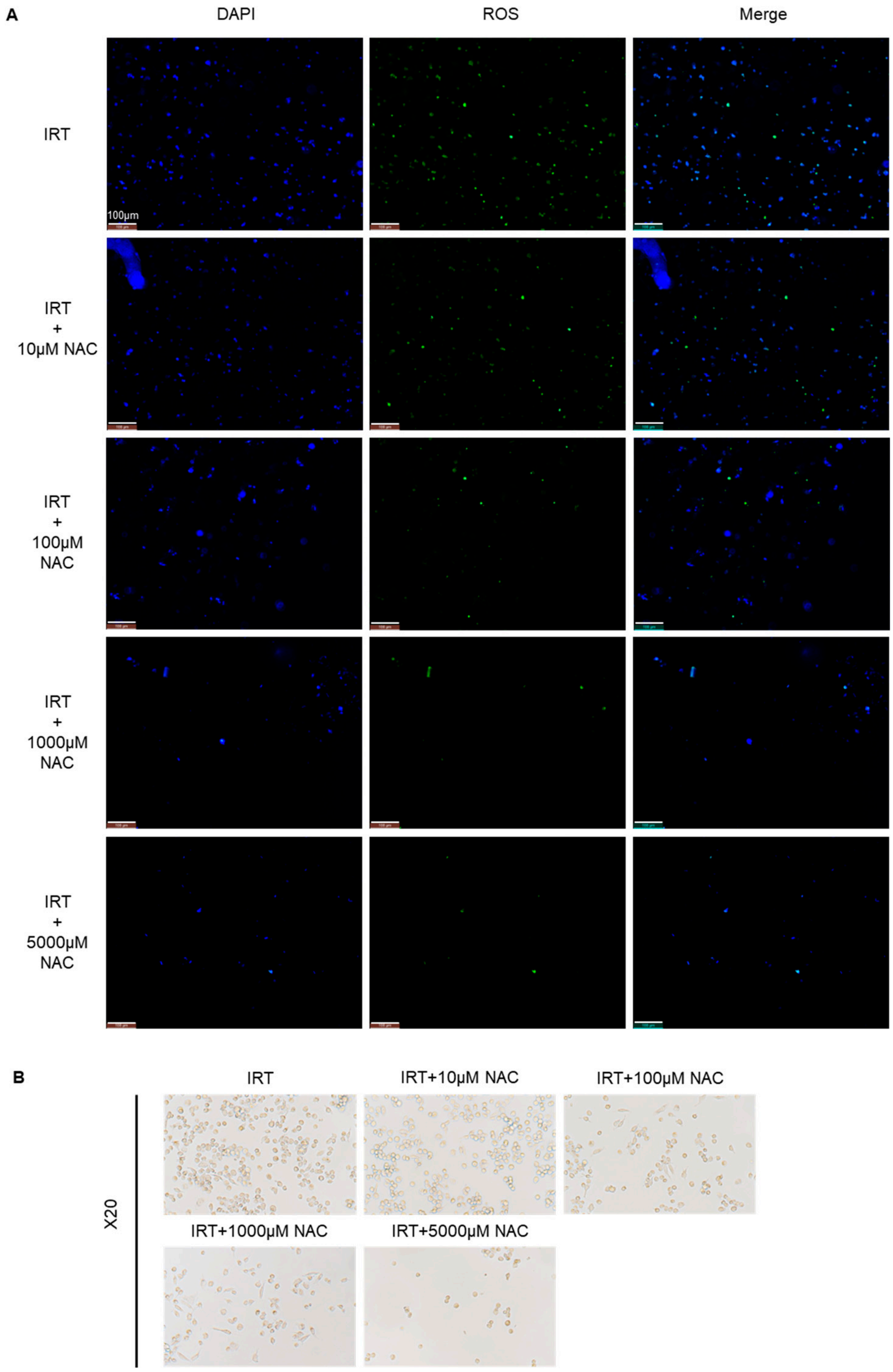


**Figure S3. Changes in intracellular ROS levels after different concentrations of NAC intervention with IRT treatment of hepatocytes.** (**A**)Primary mouse hepatocytes (3 × $10^5$) were cultured in a 24-well plate and incubated with 10 μM irinotecan and different concentration of NAC (0μM,10μM,100μM,1000μM and 5000μM) for 24 h and the levels of intercellular ROS were measured. (**B**) L02 cells (3×$10^5$) were cultured in 24-well plates with 10 μM irinotecan and different concentrations of NAC (0 μM,10 μM,100 μM,1000μM and 5000μM) for 24 h. Cell morphology and cell numbers were observed.

**Figure S4**

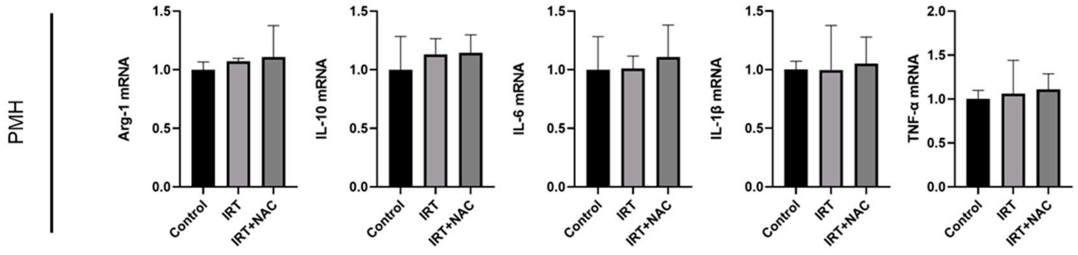


**Figure S4. Primary hepatocytes treated with IRT and NAC do not produce inflammatory factors.** We treated primary hepatocytes with irinotecan and NAC but there were no significant changes in the expression of Arg-1, IL-10, IL-6, IL-1β, and TNF-$\alpha$. Data are reported as mean ± SD, and each graph is representative of at least 3 independent experiments. Comparison of values was performed by One-way ANOVA for unpaired data. *p <0.05; **p <0.01; ***p <0.001.

**Figure S5**

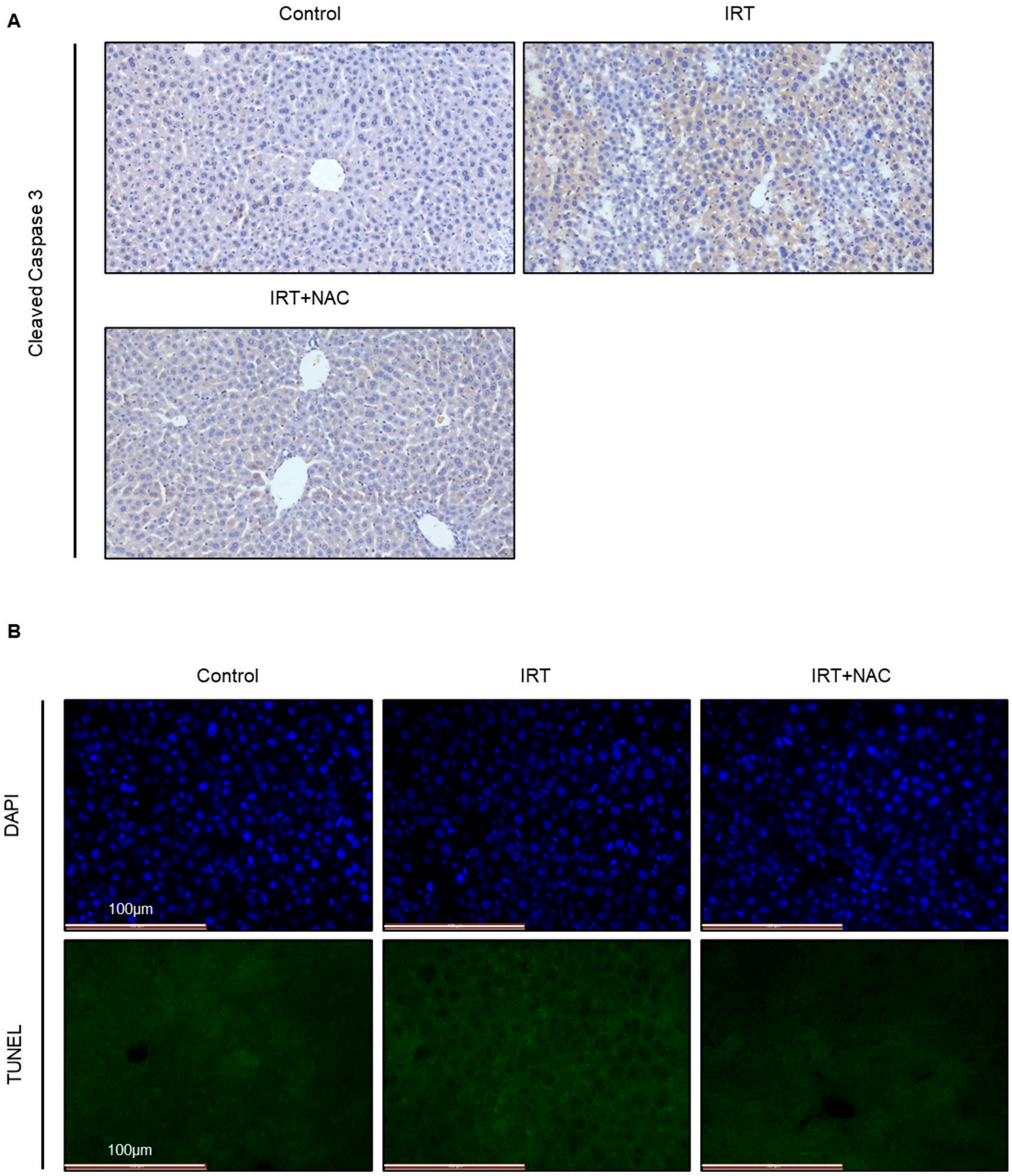


**Figure S5. Apoptosis assay under in vivo conditions.** Mice in the IRT group were injected intraperitoneally with irinotecan hydrochloride, and mice in the NAC group were simultaneously injected intraperitoneally with NAC on the following day, alternately for 2 weeks. Cleaved caspase 3 (A) level and TUNEL assay (B) were used to evaluate apoptosis in the liver.
